# LncRNA HCP5 promotes triple negative breast cancer progression as a ceRNA to regulate BIRC3 by sponging miR‐219a‐5p

**DOI:** 10.1002/cam4.2335

**Published:** 2019-06-18

**Authors:** Lihong Wang, Tian Luan, Shunheng Zhou, Jing Lin, Yue Yang, Wei Liu, Xiao Tong, Wei Jiang

**Affiliations:** ^1^ Department of Pathophysiology Medical College of Southeast University Nanjing China; ^2^ Institute of Cancer Prevention and Treatment Heilongjiang Academy of Medical Science, Harbin Medical University Harbin China; ^3^ College of Automation Engineering Nanjing University of Aeronautics and Astronautics Nanjing China; ^4^ Department of Pathology The First Affiliated Hospital of Harbin Medical University Harbin China

**Keywords:** BIRC3, competing endogenous RNA, HCP5, MiR‐219a‐5p, triple-negative breast cancer

## Abstract

Emerging evidence has suggested that long noncoding RNAs (lncRNA) involved in the development and progression of cancer. Triple negative breast cancer (TNBC) was an aggressive type of breast cancer with high rates of cancer recurrence and metastasis. The pathogenesis of TNBC is largely unknown. Recent studies suggested that lncRNA HCP5 plays an important role in carcinogenesis. The purpose of this study was to examine the function and mechanism of HCP5 in TNBC. We observed that HCP5 was upregulated in TNBC cell lines and specimens. HCP5 knockdown induced TNBC cell apoptosis, and inhibited cell proliferation and orthotopic xenograft tumor growth. RNA sequencing and antibody array suggested that HCP5 achieves its functions through regulating apoptosis pathway. Bioinformatics, luciferase and RIP experiments proved that both HCP5 and BIRC3 could competitively bind to miR‐219a‐5p. Increased BIRC3 and decreased miR‐219a‐5p were observed in TNBC tissues and cell lines. We then performed gain‐ and loss‐of‐function studies as well as rescue experiments in TNBC cells. The decrease of proliferation and migration due to HCP5 knockdown could be rescued when miR‐219a‐5p inhibitor or BIRC3 was transfected and vice versa. Our study suggested that lncRNA HCP5 promotes TNBC progression as a ceRNA to regulate BIRC3 by sponging miR‐219a‐5p. In a word, we revealed a new signaling pathway to mediate TNBC, and provided HCP5 as a new target for improving treatment of TNBC.

## 1 BACKGROUND

triple‐negative breast cancer (TNBC) is defined as estrogen receptor (ER), progesterone receptor (PR), and human epidermal growth factor receptor‐2 (HER2) negative breast cancer, which accounts for about 10%‐17% of breast cancer molecular subtypes. Due to lack of the therapeutic targets, TNBC is associated with aggressive features and poor outcomes.^1^ Thus, it is important to understand the pathogenesis of TNBC and identify the mechanisms involved in TNBC progression. Long noncoding RNAs (lncRNAs) are the RNA transcripts without protein coding ability and longer than 200 nucleotides.^2^ Emerging evidence has suggested lncRNA involved in the tumorigenesis and progression of breast cancer.^3^, ^4^ However, the regulatory roles of lncRNAs in TNBC are largely unknown.

Several lncRNAs, such as ARNILA, LINK‐A, and MALAT1, have been found dysregulated in TNBC and are considered as new therapeutic targets.^5^, ^6^, ^7^ LncRNA human histocompatibility leukocyte antigen (HLA), HLA complex P5 (HCP5), is primarily found expressed in immune system cells and had a potential role in autoimmunity.^8^ Recent studies reported that HCP5 showed aberrant expression in some human cancers. HCP5 was significantly downregulated in patients with ovarian cancer,^9^ but was considered as a susceptibility locus for HCV‐associated hepatocellular carcinoma.^10^ It has been regarded as the potential biomarker of lung adenocarcinoma^11^ and promotes the progression of follicular thyroid carcinoma via miRNAs sponge.^12^ HCP5 can also regulate malignant behavior of glioma cells through HCP5‐miR‐139‐RUNX1 feedback loop^13^ and promotes the development of cervical cancer by regulating MACC1 via suppression of miR‐15a.^14^ Recent report suggests that SP‐1 can induce HCP5 upregulation and then promote the development of osteosarcoma.^15^ Through coexpression network analysis, HCP5 was found to be associated with breast cancer prognosis.^16^ However, a possible functional role of HCP5 in TNBC remains elusive.

In this study, we found that HCP5 expression was much higher in TNBC cell lines and tumor tissues. And HCP5 knockdown promoted apoptosis, inhibited proliferation, as well as tumor growth in vivo. Moreover, the RNA sequencing and antibody array results suggested that HCP5 functioned in TNBC through regulating apoptosis pathway. Using bioinformatics methods, we predicted that HCP5 functioned as a competing endogenous RNA (ceRNA) binding to miR‐219a‐5p to regulate BIRC3.

Evidence indicates that miR‐219a‐5p is a novel tumor suppressor miRNA for many kinds of human cancers. For example, miR‐219a‐5p inhibits breast cancer cell migration and epithelial‐mesenchymal transition (EMT) by targeting MRTF‐A,^17^ inhibits epithelial ovarian cancer cells by targeting the Twist/Wnt/β‐catenin signaling pathway,^18^ inhibits nonsmall cell lung cancer by targeting HMGA2,^19^ and inhibits malignant melanoma by targeting BCL‐2.^20^

BIRC3 (Baculoviral IAP Repeat Containing 3, also known as cIAP2) is a member of inhibitor of apoptosis proteins (IAPs) family, which are major regulators that block apoptosis by preventing the activation of caspases.^21^ It can directly bind to caspases‐3, ‐7, and ‐9 and then inhibits their activation.^22^ It also can be a possible therapeutic target to modulate neurodegenerative disorders,^23^ colorectal cancer, and other malignancies.^24^ Besides acting as direct inhibitor of apoptotic pathways, BIRC3 has also been implicated in the activation of signal transduction pathways associated with malignancy.^25^ However, the mechanism regulating BIRC3 expression in TNBC remains elusive.

Here, through bioinformatics prediction and biological experiments, we identified that HCP5 could promote TNBC progression through binding to miR‐219a‐5p to activate BIRC3.

## 2 METHODS

### 2.1 TCGA dataset

We downloaded the miRNA‐seq and RNA‐SeqV2 level 3 data of breast cancer from the Cancer Genome Atlas (TCGA) database^26^ (version August, 2015). The clinical data of breast cancer samples were also obtained from TCGA database for subtype classification. We collected 1095 breast cancer samples with RNA sequencing data. We retrieved the receptor information from clinical data, the basal‐like subtype was considered as ER, PR, and HER2‐negative samples. As a result, we identified 115 basal‐like samples, in which 58 samples with mRNA, miRNA, and lncRNA expression data were retained for further analysis.

As for the prediction of ceRNA regulatory relationship, we combined the target information and the expression correlations of lncRNA, miRNA, and mRNA. First, the miRNA‐lncRNA target relationships were obtained from miRcode,^27^ which identified putative target sites based on seed complementary and evolutionary conservation. Then, the miRNA‐mRNA target relationships were obtained from targetScan.^28^ The expression correlations of miRNA‐mRNA, miRNA‐lncRNA and lncRNA‐mRNA pairs were calculated by Pearson correlation analysis. *P* < 0.05 was considered to be statistically significant.

### 2.2 Tissue samples

Breast tumor tissue microarrays (TMA) were obtained from Shanghai Outdo Biotech Co. (Shanghai, China). The TMA HBre‐Duc060CS contained 30 cases of invasive ductal carcinomas and 30 normal breast tissues from the regions around cancers. No patients received adjuvant radiotherapy, chemotherapy, or immunotherapy before surgery. The experimental protocols were approved by The Human Research Ethics Committee from Harbin Medical University.

### 2.3 Cells and culture conditions

Human breast cancer cell lines and normal human breast epithelial cell line MCF‐10A were obtained from the Shanghai Institutes for Biological Sciences, Chinese Academy of Sciences (Shanghai, China). MCF10A cell line was cultured in F12/DMEM 1:1 medium. MCF‐7 cell line was cultured in MEM medium with bovine insulin 0.01 mg/mL and sodium pyruvate 0.11 mg/mL. T‐47D and SK‐BR‐3 cell lines were cultured with DMEM medium. All cells lines were cultured with 10% fetal bovine serum (FBS) in 37°C and 5% CO_2_ incubator. MDA‐MB‐231, MDA‐MB‐468, and MDA‐MB‐453 cell lines were cultured in L‐15 medium with 10% fetal bovine serum (FBS) in 37°C incubator without CO_2_.

### 2.4 RNAscope^®^ 2.0 analysis for HCP5 RNA detection

Hybridization was done with target probes (probe symbols: a 20ZZ probe named Hs‐HCP5 targeting 576‐1623 of NR_040662.1). The detection procedures were according to the manufacturer's instructions (RNAscope^®^ 2.0 HD Reagent Kit, Advanced Cell Diagnostics, Hayward, CA, USA).

### 2.5 The LIVE/DEAD^®^ Viability/Cytotoxicity assay

Double staining with Calcein‐AM and ethidium homodimer (EthD‐1) was used to detect live and dead cells. MDA‐MB‐231 and MDA‐MB‐468 cells were transfected with HCP5 siRNA or scramble siRNA, and then growth medium was removed and cells were gently washed with PBS. Assay reagents were prepared according to the protocol, and 150 μL to each well of 96‐well plate was added. Then the plate was placed in 37°C incubator for 20 minutes. And then, the plate was detected with LB 942 TriStar^2 ^of BERTHOLD. The live cells were observed as green using a 530 nm excitation filter, while the dead cells were observed as red using a 645 nm excitation filter. The percentage of live and dead cells was calculated according to the formula of the protocol.

### 2.6 Immunohistochemical staining

The tissue sections were dried 1 hour at 60°C and then dewaxed in xylene and rehydrated through graded alcohol concentrations according to standard procedures. Antigen retrieval was performed in citrate buffer (pH 6.0) and autoclaved for 90 seconds at 121℃. After washing in PBS (3 min × 3), sections were blocked with goat serum (Boster, Wuhan, China) in room temperature for 30 minutes. Then each section was treated with BIRC3 rabbit polyclonal antibodies (bs‐5803R) (Bioss Antibodies, Inc; at a dilution of 1:200 solution) at 4℃ overnight. After washing in PBS (5 min × 3), each section was incubated with Polink‐1 HRP DAB Detection System One‐step polymer detection system for Rabbit antibody (ZSGB‐BIO, Beijing, China) at room temperature for 20 minutes. After washing in PBS (3 min × 3), the slides were counterstained with hematoxylin.

Evaluation of BIRC3 staining was performed with bright‐field light microscopy independently by two experienced pathologists, who had no knowledge of the clinicopathological information. Staining of BIRC3 protein was observed in the cytoplasm, and the tissues were divided into three groups according to expression level as follows: 0‐24, 25‐49, and ≥ 50% positive staining of tumor cells. High expression was considered as positive staining ≥50%, low expression was considered if the neoplastic cells were stained 25%‐49% and as negative if <25% of the neoplastic cells were stained. Furthermore, cases with discrepancies were re–reviewed simultaneously by the two pathologists and a senior pathologist until a consensus was reached.

### 2.7 Gene silencing by siRNA and lentivirus‐mediated transduction of shRNA

For transfection of the miRNA mimics and siRNAs, MDA‐MB‐231 (1 × 10^5^) and MDA‐MB‐468 (2 × 10^5^) cells were seeded in 6‐well plates. In the following day, they were transfected with 100 nM of miRNA mimics or 50 nM siRNA using Lipofectamine 2000 Reagent (Life Technologies). The procedure of lentivirus infection is as follows: the plate containing cells was added with appropriate amount of lentivirus in concentration gradient, followed by adding 1/1000 polybrene to enhance infection. The sequence of the miR‐219a‐5p mimics was 5′‐UGAUUGUCCAAACGCAAUUCU′ (sense) and 5′‐AAUUGCGUUUGGACAAUCAUU‐3′ (antisense). The sequence of the HCP5 siRNA was 5′‐GCAGTGTGCTTCCTTCCTT‐3′. The sequence of BIRC3 siRNA was 5′‐CAGTTCGTACATTT CTTTCAT‐3′. The sequence of the negative control (NC) siRNA was 5′‐TTCTCCGAACGTGTCACGT‐3′. These sequences were synthesized by GenePharma Co., Ltd. (Shanghai, China). Lentivirus vector LV10 was purchased from GenePharma Co., Ltd. (Shanghai, China).

### 2.8 Gene overexpression by plasmid vectors and lentivirus‐mediated transfection

Lentiviral LV5 containing full‐length HCP5 or empty vector and full‐length BIRC3‐pcDNA 3.3 vectors or empty vector were purchased from GenePharma Co., Ltd. Cells were transfected with 2 μg plasmid as well as the empty vector in Opti‐MEM medium (Invitrogen) with Lipofectamine 2000 reagent (Invitrogen) according to the manufacturer's protocol.

### 2.9 RNA extraction and quantitative reverse transcription PCR

TRIzol Reagent (Life Technologies) was used to extract total RNA according to the manufacturer's protocol. RNA was quantified and reverse transcribed into cDNA using the ReverTra Ace‐α qPCR RT Kit (Toyobo, Japan). RT‐PCR of the mature miRNAs was performed using miRcute miRNA First‐Strand cDNA Synthesis Kit (Tiangen, Beijing, China). According to the user guide of the SYBR^®^ Green Realtime PCR Master Mix (Toyobo, Japan), the qRT‐PCR amplification was done on ABI7500 Fast system. Melting curve analysis was used to monitor the specificity of the PCR products. GAPDH was used as a control. The HCP5 primers were as follows: forward, 5′‐GACTCTCCTACTGGTGCTTGGT‐3′; reverse, 5′‐CACTGCCTGGTGAGCCTGTT‐3′. The BIRC3 primers were as follows: forward, 5′‐CCAAGTGGTTTCCAAGGTGT‐3′; reverse, 5′‐TGGGCTGTCTGATGTGGATA‐3′. The GAPDH primers were as follows: forward, 5′‐CGGAGTCAACGGATTTGGTCG‐3′; reverse, 5′ ‐TCTCGCTCCTGGAAGATGGTGAT‐3′. The miR‐219a‐5p primers were as follows: 5′‐GCTGATTGTCCAAACGCAATTCT‐3′; U6 was used as a control and the primers were as follows: forward, 5′‐CTCGCTTCGGCAGCACA‐3′; reverse, 5′‐AACGCTTCACGAATTTGCGT‐3′. All of these primers were purchased from Sangon Biotech, Shanghai, China. All experiments were performed in triplicate. The qRT‐PCR results were analyzed and expressed as relative miRNA or mRNA levels of the CT (cycle threshold) value and then were converted to fold change.

### 2.10 Luciferase report assay

We constructed wild plasmids (HCP5‐wt and BIRC3‐wt) and mutant plasmids (HCP5‐mt and BIRC3‐mt) expressing the site for miR‐219a‐5p to bind with HCP5 and BIRC3 and then transfected them into HEK293T cells respectively. MiR‐219a‐5p mimics or miRNA NC were also transfected using Lipofectamine 2000 (Invitrogen, USA). Twenty‐four hours before transfection, the cells were seeded at 1.5 × 10^4^/well in 96‐well plates. The luciferase assay was performed by the dual‐luciferase reporter assay system (Promega) 48 hours after transfection. Transfection was repeated in triplicate.

### 2.11 Western blot analysis

Cells were lysed with cold RIPA buffer supplemented with 1% phenylmethyl sulfonyl fluoride and centrifuged at 12 000 × g for 30 minutes at 4°C. BCA protein assay kit (Beyotime Institute of Biotechnology) was used to determine sample protein concentrations. Equal amounts of total protein were analyzed by sodium dodecyl sulfate‐polyacrylamide gel electrophoresis (SDS‐PAGE) and electrically transferred onto a polyvinylidenedifluoride membrane (Millipore, Shanghai, China). Membranes were blocked in 5% BSA for 1 hour at room temperature and then incubated overnight at 4°C with primary antibodies (rabbit polyclonal anti‐BIRC3 antibody, 1:500, mouse monoclonal anti‐β actin, 1:2500, Bioss, Beijing;). Membranes were then washed three times with Tween‐Tris‐buffered saline and incubated with horseradish peroxidase conjugated secondary antibody at room temperature for 1 hour. Blots were visualized by an enhanced chemiluminescence (ECL, Santa Cruz Biotechnology, Dallas, TX) kit and detected by SageCapture^TM^ MiniChemi Detection Systems. Lane 1D analysis software was used to calculate the relative integrated density values.

### 2.12 Cell proliferation assays

MDA‐MB‐231 cells were seeded in 96‐well plates and transfected with HCP5, BIRC3, miR‐219a‐5p inhibitor, miR‐219a‐5p mimics and NC. MDA‐MB‐468 cells were transfected with HCP5 siRNA, BIRC3 siRNA, miR‐219a‐5p inhibitor, miR‐219a‐5p mimics and NCs. After transfection for 4, 24, 48 and 72 hours, 10 μL of the Cell Counting Kit‐8 (CCK‐8) reagent was added into each well, incubated in 37°C, 5% CO_2_ for 2 hour, and cell growth was detected by an enzyme labeling instrument at 450 nm.

### 2.13 Transwell assays

MDA‐MB‐231 cells were transfected with HCP5, BIRC3, miR‐219a‐5p inhibitor, miR‐219a‐5p mimics, and NC. MDA‐MB‐468 cells were transfected with HCP5 siRNA, BIRC3 siRNA, miR‐219a‐5p inhibitor, or scramble siRNA, respectively. At 24 hour after transfection, cells in serum‐free media were placed into the upper chamber of an insert for migration assays (8‐μm pore size, millipore) and invasion assays with Matrigel (Sigma‐Aldrich, USA). Medium containing 10% FBS was added to the lower chamber. After 24 hours of incubation, the cells that had migrated or invaded through the membrane were stained with methanol and 0.1% crystal violet, imaged, and counted using an inverted microscope (Olympus, Tokyo, Japan). The amount of cells passing through the membrane from five different fields per sample at 200 × selected in a random manner was used to determine the capacity of cell migration or invasion.

### 2.14 RNA Binding Protein Immunoprecipitation (RIP) assay

Biotinylated miR‐219a‐5p, miR‐219a‐5p‐Mut, and NC of miR‐219a‐5p (GenePharma) were transfected into MDA‐MB‐231 and MDA‐MB‐468 cells. At 48 hours after transfection, cells were harvested and lyzed. Samples were aliquoted for input. Dynabeads M‐280 Streptavidin (Invitrogen) was used to incubate the remaining samples according to the manufacturer's protocol. Beads were washed and treated in RNase‐free solutions, then incubated with equal volumes of biotinylated miR‐219a‐5p for 15 minutes at room temperature using gentle rotation. Beads with the immobilized miR‐219a‐5p fragment were incubated in 10 mmol/L ethylenediaminetetraacetate, pH 8.2 with 95% formamide for 5 minutes at 65°C. RNAs were purified and assayed by qPCR.

### 2.15 Next generation sequencing technology

The next generation sequencing was performed by Novogene (Beijing, China). A total amount of 3 μg RNA per sample was used as input material for the RNA sample preparations. RNA degradation and contamination were monitored on 1% agarose gels. RNA purity was checked using a NanoPhotometer^®^ spectrophotometer (IMPLEN, CA, USA). RNA concentration was measured using a Qubit^®^ RNA Assay Kit in a Qubit^®^ 2.0 Fluorometer (Life Technologies, CA, USA). RNA integrity was assessed using a RNA Nano 6000 Assay Kit and a Bioanalyzer 2100 system (Agilent Technologies, CA, USA). The clustering of the index‐coded samples was performed on a cBot Cluster Generation System using TruSeq PE Cluster Kit v3‐cBot‐HS (Illumia) according to the manufacturer's instructions. After cluster generation, the library preparations were sequenced on an Illumina Hiseq platform and 125 bp/150 bp paired‐end reads were generated.

We used Trimmomatic (0.38)^29^ to trim the adapter sequence and remove low‐quality reads. Default parameters were used to process the raw data to obtain clean data, and all the downstream analyses were based on the clean data. Then, we used HISAT2 (2.1.0)^30^ to map the pair‐end clean reads to the reference genome. At last, HTSeq (0.10.0)^31^ was adopted to count the reads numbers mapped to each gene. To perform differential expression analysis of HCP5 knockdown group and NC group, DESeq2 R package (1.20.0)^32^ was utilized. Genes with *P*‐value < 0.05 and |log2(Fold Change)|>1 found by DESeq2 were considered differentially expressed. Then, volcano plot was conducted by ggplot2 R package (3.0.0). To annotate the function of the differentially expressed genes, we employed DAVID^33^ online tools to perform enrichment analysis.

### 2.16 Antibody array

We use RayBio^®^ C‐Series Human Apoptosis Array 1 kit (AAH‐APO‐1‐2) to detect whether downregulated HCP5 could influence the apoptosis pathway. Membranes were blocked and incubated with Biotinylated detection antibody cocktail, then incubated with HRP‐conjugated streptavidin and detection buffers. Image was detected with chemiluminescent imaging system. And finally, we performed densitometry and analysis (Raybiotech, Inc). The experiment was repeated in triple for the mean value.

### 2.17 Xenograft mouse model

MDA‐MB‐231 cells (8 × 10^6^) and MDA‐MB‐468 (1 × 10^7^) cells stably expressing LV10‐sh‐NC or LV10‐sh‐HCP5 were subcutaneously injected into either side of flank area of 4‐week‐old female athymic nude mice (n = 6 mice per group). Tumor volumes were measured (0.5 × length×width^2^) in mice every 5 days. After 30 days, the nude mice were sacrificed, and the tumor tissues were excised and fixed in 4% paraformaldehyde solution for further study. All animal experiments were performed in the animal laboratory center of the Second Affiliated Hospital of Harbin Medical University and in accordance with the Guide for the Care and Use of Laboratory Animals published by the US National Institutes of Health (NIH publication number 85‐23, revised 1996).

### 2.18 Statistical analysis

All data were presented as means ± SEM. All experiments were repeated at least three times. Comparison of two experimental groups was evaluated by the unpaired Student's *t* test. All statistical analyses were performed by the SPSS software version 17.0. The chi‐squared test was used to compare HCP5 or BIRC3 expression between breast cancer tissues and paired normal breast tissues and the association with clinicopathologic parameters. *P* < 0.05 was considered to be statistically significant.

## 3 RESULTS

### 3.1 HCP5 expression is upregulated in TNBC cell lines and clinical TNBC samples

**Figure 1:**
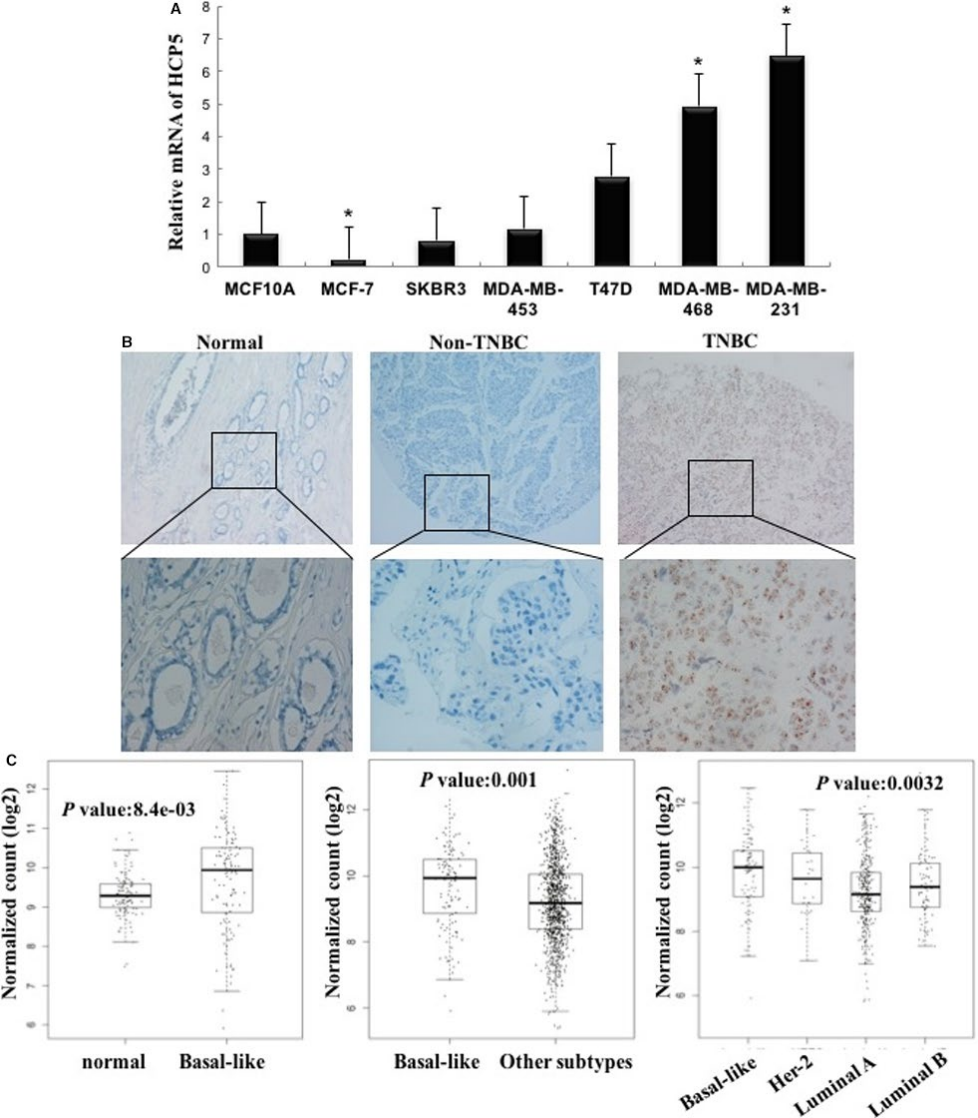
Expression of HCP5 in breast cancer cells and tissues. A, HCP5 expression was higher in TNBC cell lines than in normal breast epithelial cell line MCF‐10A and other breast cancer cell lines. ^*^ *P* < 0.05, two‐sided Student's *t* test; n = 3. B, RNA Scope detection showed that HCP5 expression was significantly upregulated in TNBC tissues than in adjacent normal tissues and other breast cancer subtype tissues (up × 100; bottom × 400). C, HCP5 expression was higher in basal‐like breast cancers than in normal breast tissues and other subtypes from TCGA database

To assess the expression of HCP5 in TNBC, we first detected the mRNA level in the human normal breast epithelial cell line MCF‐10A and breast cancer cell lines by qPCR. We found that HCP5 expression was higher in TNBC cell lines MDA‐MB‐231 and MDA‐MB‐468 than in MCF‐10A and other cell lines (Figure 1A). Then we assessed the expression of HCP5 in human breast cancer tissues, we detected HCP5 mRNA by RNA Scope^®^ 2.0 technology in 30 paired case‐control TMA. We found that HCP5 was higher in TNBC tissues than in normal breast tissues and other molecular subtypes (Figure 1B and Table S1, *P* = 0.007). The association between HCP5 expression and clinicopathologic parameters was also tested by the chi‐squared tests. The results suggested that HCP5 expression was higher in breast cancer patients with TNM III stage (Table S2, *P* < 0.001). Furthermore, we found that there were more HCP5‐positive specimens in TNBC than in other subtypes (Table S3, *P* = 0.006), which indicated that HCP5 expression was positively associated with TNBC. Our above results suggested that HCP5 upregulation may be associated with the occurrence and progression of TNBC.

To support our finding, we downloaded the Cancer Genome Atlas (TCGA) data set of 1095 clinical invasive breast cancer samples and 113 non‐tumor breast tissues. In this data set, 115 TNBC samples were included. Compared with the nontumor breast tissues, the expression of HCP5 was significantly elevated in breast cancer samples (Figure 1C). Moreover, the HCP5 expression is much higher in the basal‐like specimens than in other molecular subtypes (Figure 1C).

### 3.2 The biological function of HCP5 in TNBC cells

**Figure 2:**
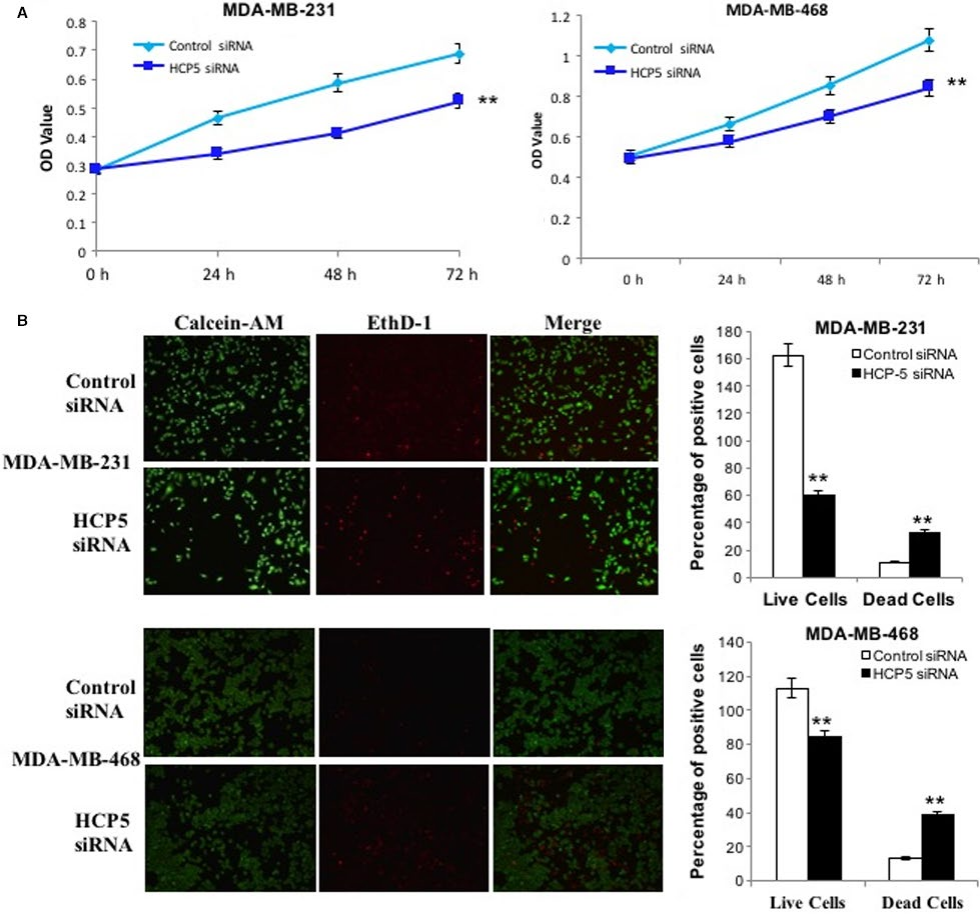
HCP5 downregulation inhibited TNBC cells proliferation and promoted apoptosis. A, CCK‐8 assays were performed to measure the proliferation of MDA‐MB‐231 and MDA‐MB‐468 cells. **P < 0.01 were calculated by using the two‐sided Student's *t* test; n = 6. B, HCP5 downregulation promoted MDA‐MB‐231 and MDA‐MB‐468 cells apoptosis as shown by Calcein‐AM/EthD‐1 staining. Green: live cells, Red: dead or dying cell (×100)

In order to test the biological functions of HCP5, we knocked down HCP5 by siRNA in MDA‐MB‐231 and MDA‐MB‐468 cells. By performing CCK‐8 assays, we found that endogenous HCP5 knockdown could significantly slower the proliferative capacity of MDA‐MB‐231 and MDA‐MB‐468 cells compared with NC cells (Figure 2A). The LIVE/DEAD^®^ assay results indicated that compared with NC group, the MDA‐MB‐231 and MDA‐MB‐468 cells transfected with HCP5 siRNA showed an apoptosis enhancement (Figure 2B).

**Figure 3:**
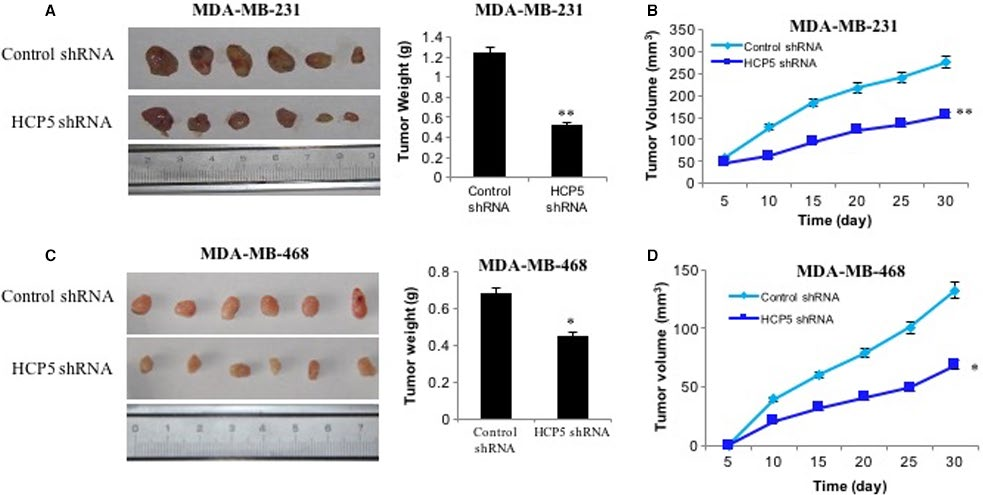
Knockdown of HCP5 inhibited breast cancer cells growth in vivo. A, Representation picture and tumor weight of tumor formation of xenograft in nude mice in lv10‐sh‐control and lv10‐sh‐HCP5 MDA‐MB‐231 cells (each group n = 6). B, Summary of tumor volume of mice which were measured in every 5 days in lv10‐sh‐control and lv10‐sh‐HCP5 MDA‐MB‐231 cells. C, Representation picture and tumor weight of tumor formation of xenograft in nude mice in lv10‐sh‐control and lv10‐sh‐HCP5 MDA‐MB‐468 cells (each group n = 6). D, Summary of tumor volume of mice which were measured in every 5 days in lv10‐sh‐control and lv10‐sh‐HCP5 MDA‐MB‐468 cells. ^**^ *P* < 0.01

Then, we investigated the in vivo activity of HCP5 in nude mice. To confirm whether HCP5 affects tumorigenesis, we examined TNBC cell lines MDA‐MB‐231 and MDA‐MB‐468 transfected with lentiviral LV10‐sh‐HCP5 sense sequence or control scramble sequence LV10‐sh‐NC. Tumors were grown for 30 days and the mice were sacrificed and tumors excised (n = 6 for each group). We found that HCP5 knockdown significantly reduced the tumor volume and weight of TNBC cell lines compared with control groups (Figure 3A,B). These data revealed that HCP5 was involved in tumorigenesis and downregulation of HCP5 inhibited TNBC cell growth both in vitro and in vivo.

To identify the pathway regulated by HCP5 in TNBC, we performed next generation sequencing and compared the RNA expression in HCP5 knockdown and NC MDA‐MB‐231 cells. The analysis of the sequencing data revealed significant changes in the RNA expression profile between the two groups. Based on *P* < 0.05 and the |log (Fold Change)|>1, 43 genes exhibited increased expression and 449 genes exhibited decreased expression in HCP5 knockdown cells (Figure S1A). We performed DAVID functional enrichment analysis using these differentially expressed gene at *P* < 0.01 (Figure S1B). According to the enrichment pathways, we supposed that HCP5 might regulate the apoptosis pathway contributing to the TNBC progression.

**Figure 4:**
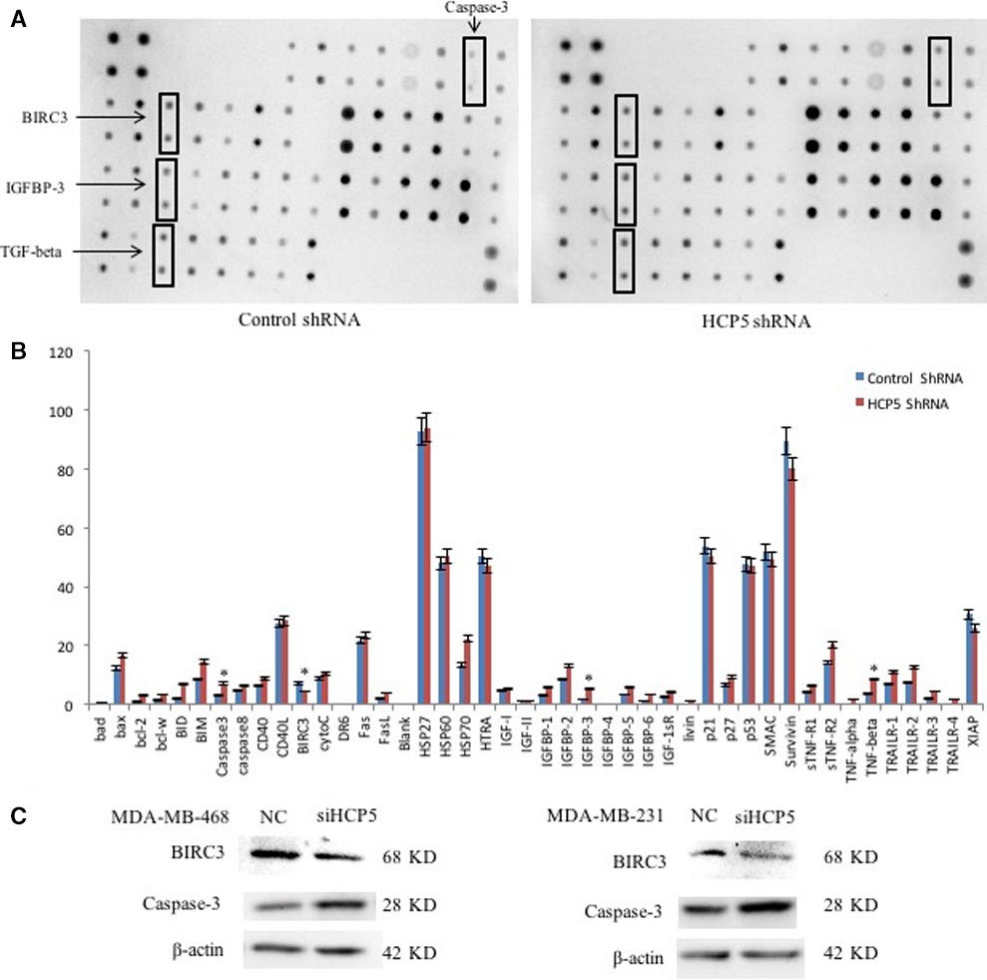
Knockdown of HCP5 activated apoptosis pathway. A and B, Representative picture of antibody array showed an obviously decreased expression of BIRC3 and increased expression of caspase‐3, IGFBP‐3 and TGF‐beta were obtained in HCP5 stable knockdown MDA‐MB‐231 cells. ^*^ *P* < 0.05, two‐sided Student's *t* test; n = 3. C, Western blot showed that the protein expression of BIRC3 was decreased while caspase‐3 was increased in HCP5 knockdown MDA‐MB‐231 and MDA‐MB‐468 cells

To evaluate the function of HCP5 in apoptosis pathway, we performed antibody array to detect the apoptosis pathway protein expression in HCP5 knockdown and NC MDA‐MB‐231 cells. As shown in Figure 4A,B, an obviously decreased expression of BIRC3 and increased expression of caspase‐3, IGFBP‐3 and TGF‐beta were obtained in HCP5 stable knockdown cells. We also demonstrated that the expression of BIRC3 decreased and its downstream caspase‐3 increased in HCP5 knockdown cells by western blot (Figure 4C).

### 3.3 HCP5 functions as ceRNA predicted by bioinformatics analysis

**Figure 5:**
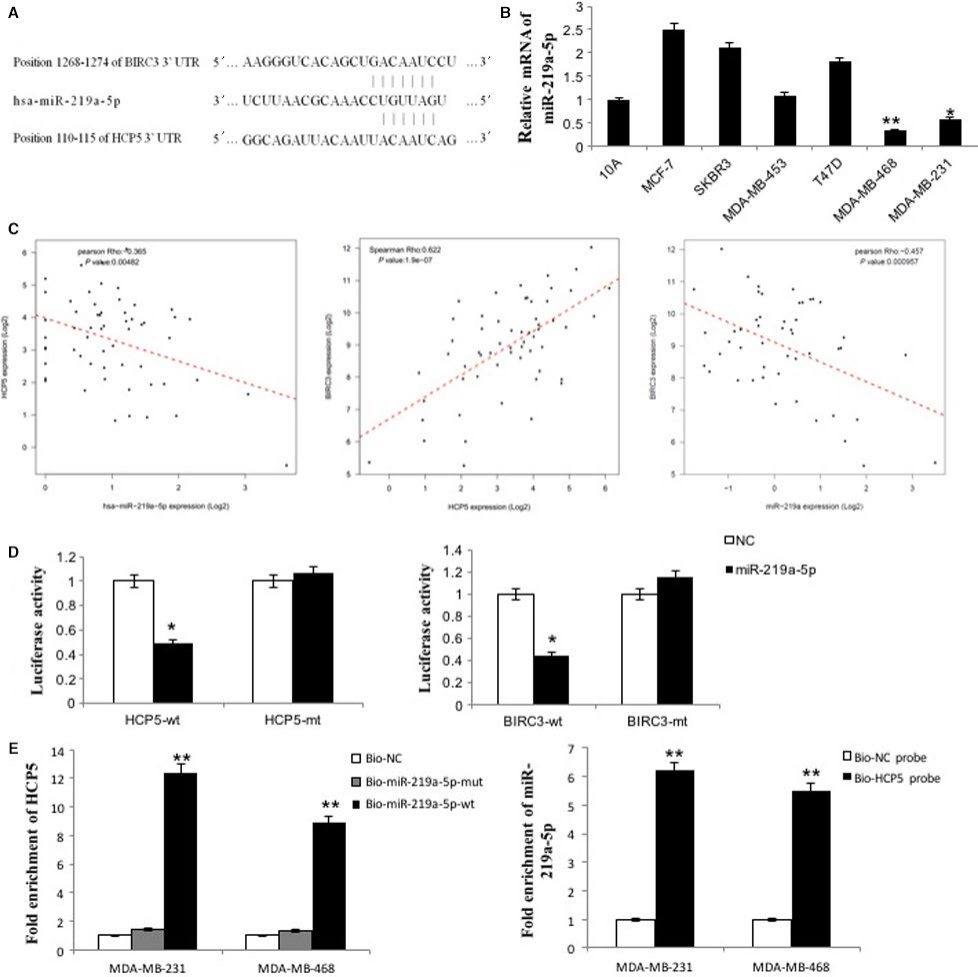
HCP5 and BIRC3 shared a common miR‐219a‐5p binding site. A, Bioinformatics analysis revealed that HCP5 and BIRC3 shared a common miR‐219a‐5p binding site. B, MiR‐219a‐5p expression was lower in TNBC cell lines than in normal breast epithelial cell line MCF‐10A and other breast cancer cell lines. ^*^ *P* < 0.05 and ^**^ *P* < 0.01. Two‐sided Student's *t* test; n = 3. C, A total of 58 basal‐like cases from TCGA database were enrolled and analyzed by Pearson correlation coefficient. The correlation between HCP5 and BIRC3 was positive in TNBC patients. And the correlations between miR‐219a‐5p and HCP5 or miR‐219a‐5p and BIRC3 were all negative. D, Luciferase activity in HEK293T cells cotransfected with miR‐219a‐5p mimics and luciferase reporters containing control vector, plasmids HCP5‐wt, HCP5‐mut, BIRC3‐wt, and BIRC3‐mut. MiR‐219a‐5p mimics reduced the luciferase activity of HCP5‐wt or BIRC3‐wt reporter vector but not that of HCP5‐mut or BIRC3‐mut reporter vector. ^*^ *P* < 0.05, two‐sided Student's *t* test; n = 3. E, QPCR was used to detect HCP5 in the sample pulled down by biotinylated miR‐219a‐5p. And qPCR was used to detect miR‐219a‐5p in the sample pulled down by biotinylated HCP5 probe. (*n* = 3, each group). ^**^ *P* < 0.01

Then we investigated how HCP5 regulates the apoptosis pathway. Since the function research of lncRNA is mainly focused on ceRNA, we used bioinformatics methods to analyze whether HCP5 functions as ceRNA to regulate the apoptosis pathway in TNBC. First, we found 67 miRNAs that could target HCP5 from miRcode, in which 60 miRNAs expressed in TNBC samples. Second, we calculated the Pearson correlation coefficient between HCP5 and miRNAs, only miR‐219a‐5p was negatively correlated with HCP5. To evaluate the apoptosis genes targeted by miR‐219a‐5p, we analyzed whether the 43 genes listed in apoptosis antibody array could be targeted by miR‐219a‐5p. As a result, 6 apoptosis related genes were confirmed. Next, the correlation of miRNA‐mRNA pairs was calculated by Pearson correlation analysis, we found that BIRC3 and FASL were negatively correlated with miR‐219a‐5p. And the expression of HCP5 and BIRC3 or FASL was positively correlated. But we abandoned FASL because there was no significant change of FASL expression after HCP5 downregulation in qPCR and antibody array experiments. The conserved binding sites for miR‐219a‐5p on both HCP5 and the 3′‐UTR of BIRC3 mRNA were shown in Figure 5A. MiR‐219a‐5p can complementarily bind to the HCP5 sequence between 110 bp and 115 bp, and its sequence is also complementary to the 3′‐UTR sequence of BIRC3 mRNA between 1268 bp and 1274 bp. Furthermore, we detected the miR‐219a‐5p expression in breast cancer cell lines and found that the expression of miR‐219a‐5p was lower in TNBC MDA‐MB‐231 and MDA‐MB‐468 cell lines than in MCF‐10A and other breast cancer cell lines (Figure 5B).

To validate our results, a total of 58 basal‐like cases with HCP5, miR‐219a‐5p and BIRC3 expression data from TCGA database were enrolled in this study and analyzed by Pearson correlation coefficient. As shown in Figure 5C, there were positive correlation between HCP5 and BIRC3 in breast cancer patients (*R* = 0.625, *P* < 0.001). Moreover, the correlations between miR‐219a‐5p and HCP5 (*R *= −0.374, *P* = 0.004) and miR‐219a‐5p and BIRC3 (*R *= −0.343, *P* = 0.008) were all negative. In summary, we speculated that HCP5 could function as ceRNA to regulate the expression of BIRC3 through harboring the miR‐219a‐5p target site in TNBC.

### 3.4 HCP5 functions as ceRNA proved by experiments

To further study the ceRNA mechanism of HCP5, a dual‐luciferase reporter assay was used to test whether HCP5 and BIRC3 are targets of miR‐219a‐5p. The luciferase assay showed that the luciferase activity was reduced in HEK293T cells that were cotransfected with miR‐219a‐5p and BIRC3‐wt or HCP5‐wt but was no change in HEK293T cells containing HCP5‐mut or BIRC3‐mut (Figure 5D). The results confirmed that miR‐219a‐5p targeting both BIRC3 and HCP5.

Then, we performed RNA immunoprecipitation (RIP) experiments to further investigate the potential direct binding between lncRNA HCP5 and the miRNAs. As shown in Figure 5E, HCP5 was pulled down by miR‐219a‐5p, but the miR‐219a‐5p mutation group was unable to pull down HCP5, meaning that recognition between HCP5 and miR‐219a‐5p was specific both in MDA‐MB‐231 and in MDA‐MB‐468 cells. We also used an inverse pull‐down assay to investigate whether HCP5 could pull down miR‐219a‐5p using a biotin‐labeled specific HCP5 probe. The qPCR results suggested that miR‐219a‐5p could recognize the HCP5 probe not the NC probe.

Next, we transfected miR‐219a‐5p mimics into MDA‐MB‐231 and MDA‐MB‐468 cell lines and qPCR analysis revealed that miR‐219a‐5p overexpression could suppress both HCP5 and BIRC3 mRNA expression (Figure S2A). According to the ceRNA hypothesis, knockdown HCP5 will result in freeing of miR‐219a‐5p, and miR‐219a‐5p will target BIRC3 mRNA and trigger the downregulation of BIRC3. Thus, we detected whether downregulation of HCP5 would influence miR‐219a‐5p and BIRC3 expression. We transfected HCP5 siRNA into MDA‐MB‐231 and MDA‐MB‐468 cells to knockdown HCP5, then qPCR revealed that the expression of miR‐219a‐5p was increased and the expression of BIRC3 was decreased. While the miR‐219a‐5p was downregulated and the BIRC3 was upregulated, when we overexpressed HCP5 in MDA‐MB‐231 and MCF‐7 cells (Figure S2B and S2C). The results suggested that HCP5 promoted TNBC progression as a ceRNA pattern through upregulating BIRC3 by sponging miR‐219a‐5p.

### 3.5 BIRC3 expression is upregulated in TNBC cell lines and clinical samples

**Figure 6:**
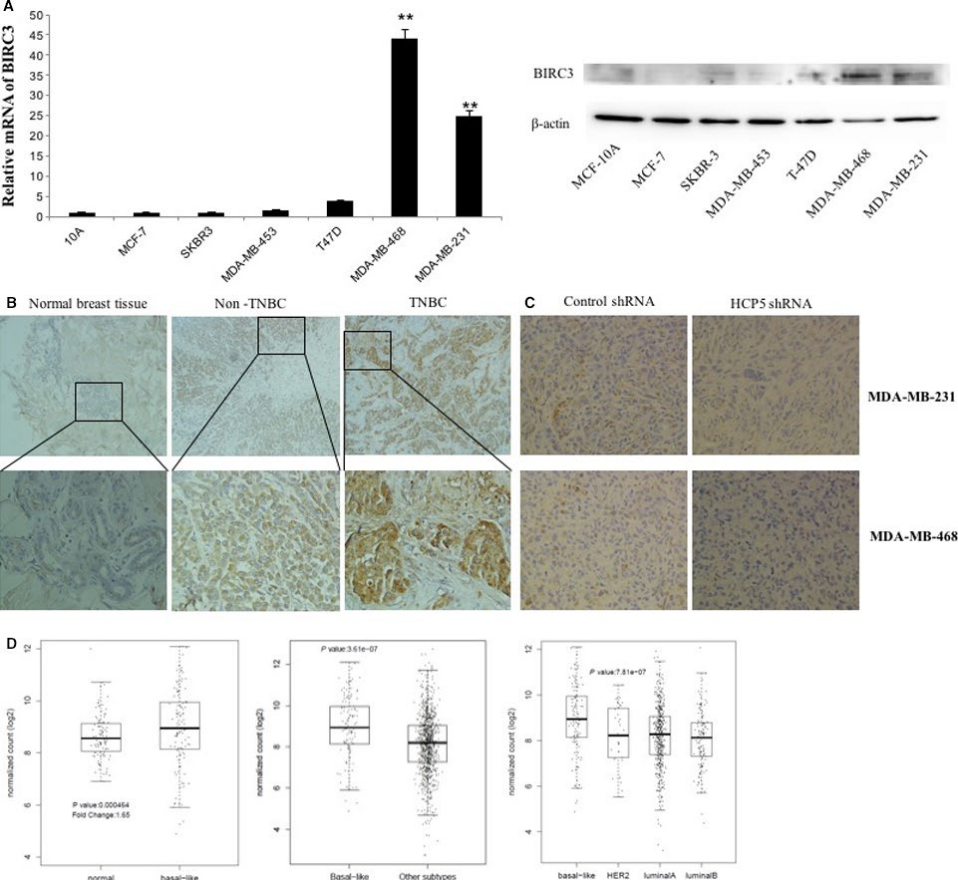
Expression of BIRC3 in breast cancer cells and tissues. A, BIRC3 mRNA and protein expression were higher in TNBC cell lines than in normal breast epithelial cell line MCF‐10A and other breast cancer cell lines. ** *P* < 0.01, two‐sided Student's *t* test; n = 3. B, IHC showed that BIRC3 expression was significantly upregulated in TNBC tissues than in adjacent normal tissues and other breast cancer subtype tissues (up × 100; bottom × 400). C, IHC analysis of xenografted tumor tissues revealed that BIRC3 expression was decreased in the HCP5 knockdown groups (×400). D, BIRC3 expression was higher in basal‐like breast cancers than in normal breast tissues and other subtypes from TCGA database

We detected BIRC3 mRNA and protein level in breast cancer cell lines by qPCR and western blot. The results suggested that BIRC3 expression was upregulated in MDA‐MB‐231 and MDA‐MB‐468 than in MCF‐10A and other breast cancer cell lines (Figure 6A). Then we assessed the expression of BIRC3 in human breast cancer tissues by immunohistochemical staining (IHC) in TMA. We found that BIRC3 was higher in TNBC tissues than in normal breast tissues and other molecular subtypes (Figure 6B and Table S4). The association between BIRC3 expression and clinicopathologic parameters was also tested by the chi‐squared tests. We found there were more BIRC3‐positive specimens in TNBC than in other subtypes (Table S5). And further we found that there were 10 samples with BIRC3 high expression in 12 HCP5‐positive samples and seven samples with BIRC3 high expression in 18 HCP5 negative samples (Table S6), which suggested the expression of HCP5 and BIRC3 was positive association. Correspondingly, an immunostaining analysis of xenografted tumor tissues revealed that BIRC3 expression was also decreased in the HCP5 knockdown group (Figure 6C).

Then the TCGA data set was analyzed. We found that the expression of BIRC3 was significantly elevated in breast cancer samples than in normal breast tissues. Similar with HCP5, the BIRC3 expression was also higher in the basal‐like specimens than in other molecular subtypes (Figure 6D).

### 3.6 Rescue studies prove HCP5 functions as a ceRNA pattern in TNBC

**Figure 7:**
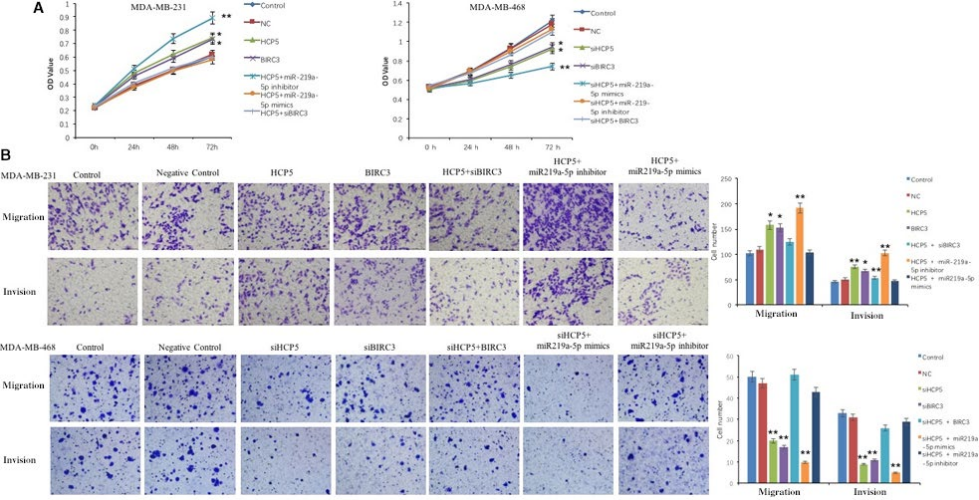
The rescue effects of miR‐219a‐5p and BIRC3 on breast cancer cells proliferation, migration and invasion. A, CCK8 assay to evaluate the rescue effect of miR‐219a‐5p inhibitor and BIRC3 on MDA‐MB‐468 cells proliferation after HCP5 knockdown and the rescue effect of miR‐219a‐5p mimics and BIRC3 siRNA on MDA‐MB‐231 cells proliferation after HCP5 overexpression. B, Transwell assay to detect the rescue effect of miR‐219a‐5p and BIRC3 on MDA‐MB‐468 or MDA‐MB‐231 cells migration and invasion after HCP5 knockdown or overexpression.^ *^ *P* < 0.05 and ^**^ *P* < 0.01, compared with control group. Two‐sided Student's *t* test; n = 3

In order to test the biological functions of lncRNA HCP5, we performed gain‐ and loss‐of‐function studies in TNBC cells. We stably knocked down HCP5 using lentiviral LV10‐sh‐HCP5 in MDA‐MB‐468 cell and overexpressed it in MDA‐MB‐231 cells by lentiviral LV5‐HCP5. We investigated the role of HCP5 and BIRC3 in proliferation using a CCK8 assay. The results showed that endogenous HCP5 or BIRC3 knockdown could significantly slower the proliferative capacity of MDA‐MB‐468 cells compared with parallel stable cell lines containing LV10‐sh‐NC. The proliferation decrease due to HCP5 knockdown could be rescued when miR‐219a‐5p inhibitor or BIRC3 was transfected while the proliferation decrease could be aggravated by miR‐219a‐5p mimics (Figure 7A). Next, we evaluated TNBC cell migration and invasion using transwell‐based assays. As shown in Figure 7B, HCP5 or BIRC3 knockdown could significantly decrease the migration and invasion ability of MDA‐MB‐468 cells compared with NC cells. And this inhibition caused by HCP5 downregulation could again be partially rescued by miR‐219a‐5p inhibitor or BIRC3 and aggravated by miR‐219a‐5p mimics.

To further support these results, we overexpressed HCP5 or BIRC3 in MDA‐MB‐231 cells. The CCK‐8 assays showed that the proliferation ability of HCP5 or BIRC3 overexpression cells was significantly higher than in NC cells. HCP5 or BIRC3 upregulated cells also showed increased migration and invasion ability compared with NC cells. The increase ability caused by HCP5 overexpression could be eliminated by miR‐219a‐5p mimics or BIRC3 siRNA and promoted by miR‐219a‐5p inhibitor (Figure 7A,B).

## 4 DISCUSSION

Evidence have indicated that lncRNAs are essential regulators involved in cancer pathogenesis.^21^, ^22^ But the function of lncRNAs in TNBC is still largely unknown. Recently, the abnormal expression of lncRNA HCP5 is reported in many cancers.^10^, ^12^, ^13^, ^14^ Our studies revealed that HCP5 was highly upregulated in TNBC cell lines and TNBC tumor tissues by qPCR and RNA Scope technology. To understand the biological function of HCP5 in TNBC, we knocked down the endogenous expression of HCP5 in TNBC cell lines. We found that HCP5 downregulation increased cell apoptosis, inhibited proliferation and tumor growth in vivo. We speculated that upregulation of HCP5 in TNBC may promote tumor aggressive. Further, we did next generation sequencing to identify the differentially expressed genes or pathways regulated by HCP5. There were 43 upregulated genes and 449 downregulated genes in HCP5 knockdown cells. The enrichment analysis suggested that the apoptosis pathway was involved. Then we did antibody array to detect the protein expression in apoptosis pathway. The data suggested most of the apoptosis‐associated protein expression was upregulated, but only caspase‐3, IGFBP‐3 and TGF‐beta were increased with statistical significance in HCP5 knockdown cells. The mechanism of HCP5 regulated IGFBP‐3 and TGF‐beta in TNBC should be studied in the future. Moreover, IAP family members BIRC3, survivin, and XIAP were all decreased in HCP5 downregulation cells, but only the changes of BIRC3 is statistically significant. Inhibition of apoptotic process is a key factor in the progression of tumors toward malignancy. And IAPs are critical proteins that regulate apoptosis.

To further understand how HCP5 regulated the apoptosis genes in TNBC, we analyzed the possible regulatory mechanism using bioinformatics methods. The prediction showed that HCP5 could function as a ceRNA binding to miR‐219a‐5p to regulate BIRC3. Then, the binding between miR‐219a‐5p and HCP5 or BIRC3 were verified by luciferase and RIP technology. MiR‐219a‐5p has been reported as a tumor suppressing miRNA for many cancers.^17^, ^19^, ^20^ We found miR‐219a‐5p expression is downregulated in TNBC cells, and overexpression of miR‐219a‐5p could inhibit both HCP5 and BIRC3 mRNA level. Further experiments suggested that HCP5 overexpression decreased miR‐219a‐5p but increased BIRC3 mRNA level and vice versa. The relationships between HCP5 and BIRC3 expression were positive, and the relationships between miR‐219a‐5p and HCP5 or BIRC3 expression were negative. All above data suggested that HCP5 was a ceRNA combined with miR‐219a‐5p to regulate BIRC3 in TNBC.

BIRC3 is an inhibitor of apoptosis and as potential oncogene in many cancers through inhibiting apoptosis and facilitating cancer cell survival.^34^, ^35^ A potent small‐molecule antagonist of IAP proteins has been discovered as clinical candidate for cancer treatment.^36^ In this study, we found BIRC3 expression was upregulated in TNBC cells and tumor tissues, which consistent with TCGA database analysis and other reports.^26^ The BIRC3 expression was also decreased in HCP5 knockdown cells and xenografted tumor tissues. We then performed gain‐ and loss‐of‐function studies and rescue experiments in TNBC cells. The proliferation and migration declined due to HCP5 downregulation could be rescued when miR‐219a‐5p inhibitor or BIRC3 was transfected. All these results indicated that HCP5 competitively sponging miR‐219a‐5p leading to activation of BIRC3 in apoptosis signaling pathway promoted TNBC progression.

In summary, the data show that lncRNA HCP5 is significantly upregulated in TNBC and promotes TNBC progression as a ceRNA. Furthermore, our study highlights the role of HCP5 and demonstrates that targeting HCP5 could be a promising therapeutic strategy in TNBC patients. However, due to the limited samples of the present study, larger cohorts are required to validate the association between HCP5 and the clinicopathologic parameters of TNBC patients. And the other pathways regulated by HCP5 should also be considerate in the following research.

## CONFLICT OF INTEREST

The authors declare no competing financial interests.

## AUTHOR CONTRIBUTIONS

LW and TL performed most of the experiments, analyzed data, and helped in drafting the manuscript. SZ did bioinformatics and RNA‐Seq analysis. YY performed xenograft tumor experiments in mice. JL performed histopathological examination. WL and XT reviewed the immunohistochemical staining tissue sections. WJ and LW designed and supervised the study and wrote the manuscript. All authors read and approved the final manuscript.

## ETHICS APPROVAL

The protocols for animal experiments were approved by the Animal Care and Use committee of the Second Affiliated Hospital of Harbin Medical University.
