# Supplementary figures and images for: LncRNA HCP5 promotes triple negative breast cancer progression as a ceRNA to regulate BIRC3 by sponging miR‐219a‐5p
Source: Cancer Med. 2019 Jun 18;8(9):4389–403. doi: 10.1002/cam4.2335 (PMC6675706; doi:10.1002/cam4.2335)

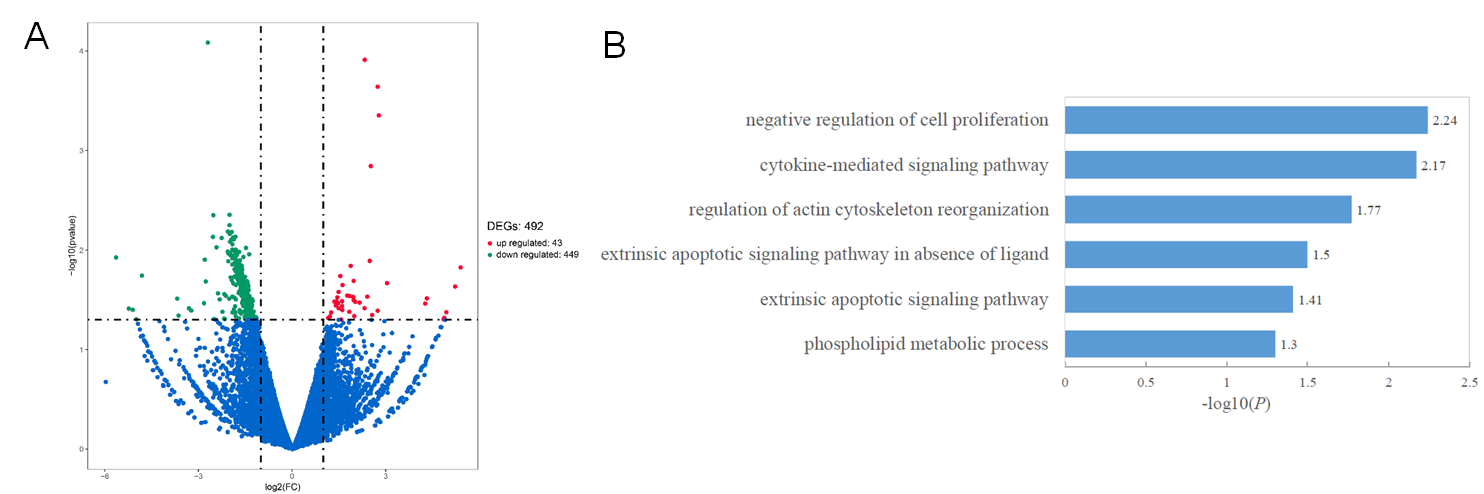

Supplement: Supplementary file 1 [file CAM4-8-4389-s001.tif]

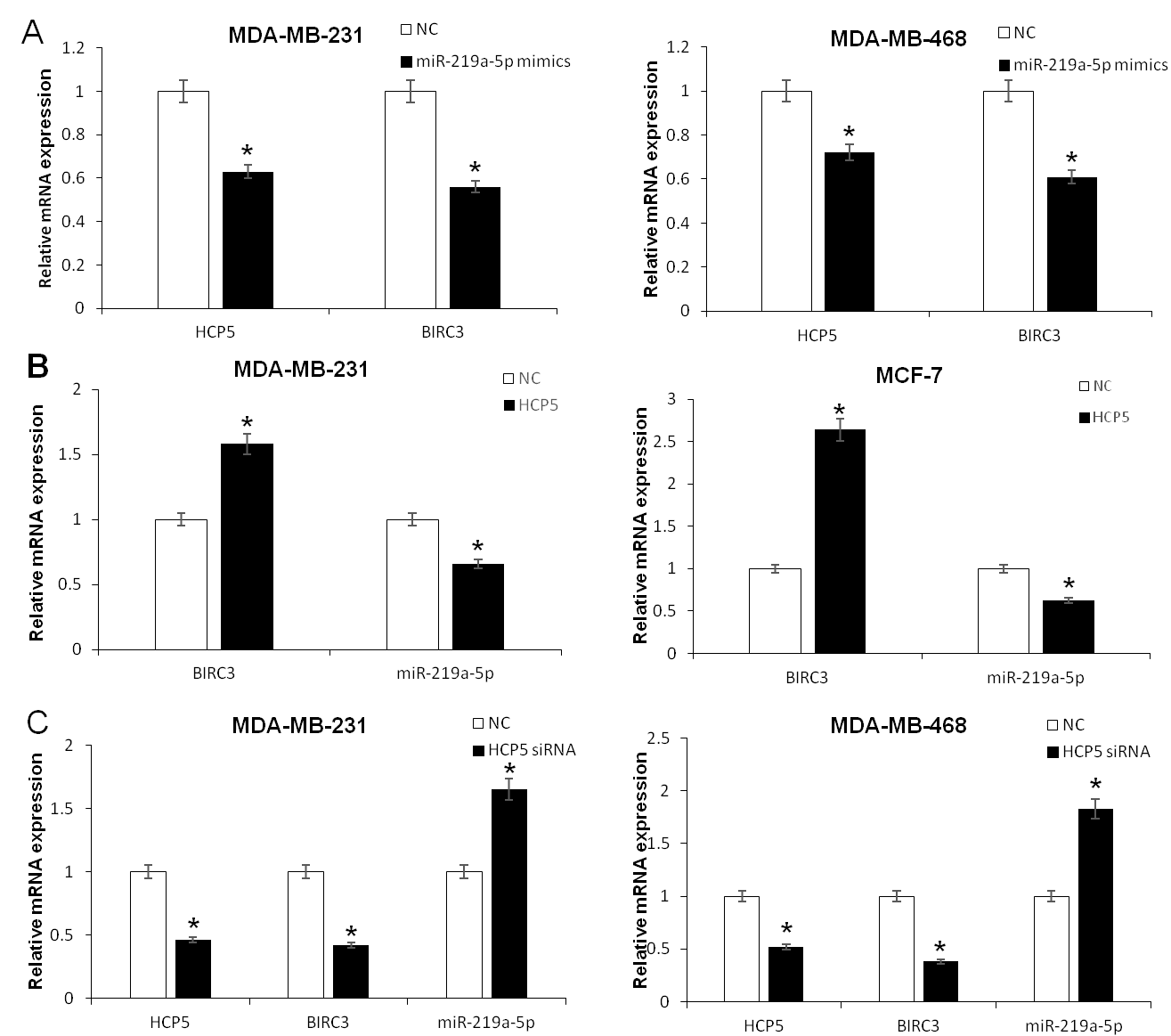

Supplement: Supplementary file 2 [file CAM4-8-4389-s002.tif]
