# Supplementary material for: LncRNA HCP5 promotes triple negative breast cancer progression as a ceRNA to regulate BIRC3 by sponging miR‐219a‐5p
Source: Cancer Med. 2019 Jun 18;8(9):4389–403. doi: 10.1002/cam4.2335 (PMC6675706; doi:10.1002/cam4.2335)
Supplement: Supplementary file 3 [file CAM4-8-4389-s003.doc]

Table S1.The expression of HCP5 in paired breast cancer tissues

|  | HCP5 | | *p* |
| --- | --- | --- | --- |
|  | - | + |  |
| normal tissues | 27 | 3 | 0.007 |
| cancer tissues | 18 | 12 |  |

Table S2. Association between HCP5 expression with TNM stage of breast cancer patients

| TNM stage | - | + | *p* |
| --- | --- | --- | --- |
| І, П | 17 | 3 | <0.001 |
| III | 1 | 9 |

Table S3. Association between HCP5 expression with subtypes of breast cancer patients

| Subtypes | - | + | *p* |
| --- | --- | --- | --- |
| TNBC | 2 | 7 | 0.006 |
| Other subtypes | 16 | 5 |

Table S4. The expression of BIRC3 in paired breast cancer tissues

|  | BIRC3 | | *p* |
| --- | --- | --- | --- |
|  | Low expression | High expression |  |
| normal tissues | 21 | 9 | 0.037 |
| cancer tissues | 13 | 17 |  |

Table S5. Association between BIRC3 expression with subtypes of breast cancer patients

| Subtypes | Low expression | High expression | *p* |
| --- | --- | --- | --- |
| TNBC | 4 | 12 | 0.030 |
| Other subtypes | 9 | 5 |

Table S6. The expression of BIRC3 in HCP5 positive and negative breast cancer tissues

| HCP5 | BIRC3 | | | *p* |
| --- | --- | --- | --- | --- |
|  | Low expression | High expression | |  |
| - | 11 | | 7 | 0.016 |
| + | 2 | | 10 |  |
